# Prevalence and associated factors of Hepatitis C virus and human immunodeficiency virus infections among voluntary counseling and testing clients attending private health facilities in Bahir Dar city, North West Ethiopia 2014

**DOI:** 10.1186/s13104-019-4727-9

**Published:** 2019-10-25

**Authors:** Zena Ameha, Senait Tadesse, Abate Assefa, Belay Tessema

**Affiliations:** 1 Amhara Public Health Institute, Bahir Dar, Ethiopia; 2 Medical Laboratory Science Department, Health Science College, Bahir Dar University, Bahir Dar, Ethiopia; 3 University of Gondar Colleges of Medicine and Health Sciences (CMHS), Gondar, Ethiopia

**Keywords:** Hepatitis C virus, Human immunodeficiency virus, VCT, Ethiopia

## Abstract

**Objective:**

Although incredible progress has been made in treatment and prevention of Hepatitis C virus and human immunodeficiency virus infections, the epidemic continues to spread in developing nations. The information on the prevalence and risk factors of Hepatitis C virus and human immunodeficiency virus infections among voluntary counseling and testing clients in Ethiopia is limited. Hence, the study aimed to assess the prevalence and associated factors of Hepatitis C virus and human immunodeficiency virus infections among voluntary counseling and testing clients attending private health facilities in Bahir Dar city.

**Result:**

A total of 382 study participants with the mean age of 25.43 years (SD = ± 6.87) were enrolled. Overall, 14 (3.7%) and 8 (2.1%) voluntary counseling and testing clients were positive for human immunodeficiency virus and Hepatitis C virus respectively. All Hepatitis C virus antibody positive individuals were males (3.8%). The sero-prevalence of Hepatitis C virus was significantly associated with the age group 41–50 years old (AOR = 65.65; 95% CI 4.57–943). Married study participants were also significantly associated with HIV infection (AOR = 7.92, 95% CI 1.32–47.31).

## Introduction

Hepatitis C virus (HCV) and human immunodeficiency virus (HIV) infections remain a major cause of health problem worldwide, with significant overlap in the geographical areas and most affected populations [1]. They share similar transmission routes including sexual, blood–blood contact, and injecting drug usage. Although tremendous progress has been made in treatment and prevention of HIV infections, the epidemic continues to spread in developing nations. In 2012, of all new HIV infections about 70% live in sub-Saharan Africa) [2]. Co-infection with HIV and HCV is now a major public health concern worldwide, owing both to its high prevalence and to interactions between the two diseases in terms of their diagnosis, natural course of diseases, and treatment [3, 4]. In co-infected patients, HIV weakens the immune response to HCV infection. The rate of progression of hepatitis C infection to end stage liver disease is accelerated in the presence of HIV infection and HCV infection increases risk of antiretroviral associated liver toxicity and death in HIV patients [5].

Ethiopia is one of African countries hardly affected by the HIV epidemic. According to the 2011 Ethiopian Demography Health Survey (EDHS) data, the overall prevalence of HIV infection among the general population was estimated 1.5% [6]. A previous population-based survey had reported 2% prevalence of HCV infection in the country [7]. Currently, HCV screening practice is rarely implemented in the country because of the high cost of diagnostic testing and treatment services.

Accurate estimate of the prevalence and associated factors of these viruses in a particular population is very much important to plan effective preventive strategies. Therefore this study was conducted to investigate the burden and associated risk factors contributing to HCV and HIV infections among voluntary counseling and testing (VCT) clients attending private health facilities.

## Main text

### Methods

A cross-sectional study was conducted among 382 VCT clients of private health facilities in Bahir Dar city from March to April 2014. Study participants were selected by systematic random sampling method from consecutive VCT clients attending four private health facilities during the study period.

Socio-demographic and potential risk factors were collected using structured questionnaires with face to face interview. 3 ml of Blood samples were collected from each study participants and HIV infection among VCT clients was determined by anti-HIV antibody test (rapid test currently used in national algorithm). KHB (Shanghai Kehua Bio-engineering Co. Ltd., China) was used for the first screening and positive samples were re-tested with STAT-PACK (Chembio HIV 1/2 STATPAK™ Assay, CHEMBIO DIAGNOSTIC SYSTEMS, INC., MEDFORD, NY, USA) and HCV infection among VCT clients was determined by rapid anti-HCV test (the advanced quality^™^ Assay, In Tec PRODUCTS, INC, China) which has sensitivity of 100% and specificity of 97–99%. The test works based on principle of immunochromatograph.

Written informed consent was obtained from each study participant. Official permission was obtained from each private health facility included in the study. Confidentiality was maintained at each level of the study.

After data were checked for completeness and consistencies, they were entered and analyzed using Statistical Software for social package (SPSS) version 20.

Binary logistic regression analysis was done to determine the association between socio-demographic and other potential risk factors and outcome variables. All variables with a P ≤ 0.2 in the bivariate analysis were included in the multivariate logistic regression model. Odds ratio (OR) at 95% confidence interval (CI) was computed to measure the strength of association. P-value < 0.05 was considered as significant.

### Result

#### Socio-demographic characteristics

A total of 382 VCT clients were participated in this study. Of these, 55.2% were males. The mean age of the study subjects was 25.4 years with a standard deviation of ± 6.87 years. Most of the clients 79.1% were living in urban setting. In terms of religion, 77.2% of study participants were Christian. Thirty nine (10.2%) of the study participants had unable to read and write. The majority of study participants 72.8% were unmarried.

#### Prevalence of HIV and HCV infections

Prevalence of HIV and HCV infections were 14 (3.7%) and 8 (2.1%), respectively. No HIV/HCV co-infection was found in this study. The prevalence of HIV was similar between females (3.5%) and in males (3.8%). The highest proportion of HIV infection was found among age group of 41–50 years old (17.6%) and married (13.3%) study participants. On the other hand, all of HCV antibody positive study participants were males (3.8%). The rates of HCV infection were higher in the age group 41–50 years old (23.5%), farmers (4.1%) and divorced/widowed individuals (6.8%) study participants (Table 1).

**Table 1:** Prevalence of HIV and HCV among VCT clients attending private health facilities in Bahir Dar city, from March to April 2014

Variables	HIV status		HCV status	
	Positive n (%)	Negative n (%)	Positive n (%)	Negative n (%)
Sex				
Female	6 (3.5%)	165 (96.5%)	0 (0%)	171 (100%)
Male	8 (3.8%)	203 (96.2%)	8 (3.8%)	203 (96.2%)
Age (years)				
14–20	0 (0%)	105 (100%)	0 (0%)	105 (100%)
21–30	6 (2.8%)	206 (97.2%)	3 (1.4%)	209 (98.6%)
31–40	5 (10.4%)	43 (89.5%)	1 (2.1%)	47 (97.9%)
41–50	3 (17.6%)	14 (82.4%)	4 (23.5%)	13 (76.5%)
Occupation				
Student	0 (0%)	61 (100%)	0 (0%)	61 (100%)
Farmer	0 (0%)	49 (100%)	2 (4.1%)	47 (95.9%)
Merchant	2 (1.5%)	134 (98.5%)	3 (2.2%)	133 (97.8%)
Governmental employed	8 (10.8%)	66 (89.2%)	2 (2.7%)	72 (97.3%)
Other	4 (6.5%)	58 (93.5%)	1 (1.6%)	61 (98.4%)
Marital status				
Single	2 (0.7%)	276 (99.3%)	4 (1.4%)	274 (98.6%)
Married	8 (13.3%)	52 (86.7%)	1 (1.7%)	59 (98.3%)
Divorced/widowed	4 (9.1%)	40 (90.9%)	3 (6.8%)	41 (93.2%)
Religion				
Christian	14 (4.7%)	281 (95.3%)	6 (2%)	289 (98%)
Muslim	0 (0%)	87 (100%)	2 (2.3%)	85 (97.7%)
Education level				
Unable to read and write	1 (2.6%)	38 (97.4%)	3 (7.7%)	36 (92.3%)
Grade (1–8th)	2 (1.4%)	136 (98.6%)	2 (1.4%)	136 (98.6%)
Grade (9–12th)	3 (2.6%)	112 (97.4%)	1 (0.9%)	114 (99.1%)
Above grade 12th	8 (8.9%)	82 (91.1%)	2 (2.2%)	88 (97.8%)
Residence				
Urban	12 (4%)	290 (96%)	6 (2.0%)	296 (98%)
Rural	2 (2.5%)	78 (97.5%)	2 (2.5%)	78 (97.5%)

#### Risk factors for HCV and HIV infections

During bivariate analysis, No of sexual partner, History of tattooing, age and marital status of the participants were significant association with HIV. But in multivariate analysis only married study subjects had significantly associated with HIV infection. The odds of married study subjects (AOR = 7.92, 95% CI 1.32–47.31, P = 0.023) was eight times higher risk for HIV infection as compared with unmarried participants. However, HIV sero-positivity was not associated with a history of blood transfusion, surgery, sexual transmitted diseases, hospital admission and sharing of sharp materials (Table 2).

**Table 2:** Bivariate and multivariate analysis of risk factors for HIV infection among VCT clients attending Private health facilities in Bahir Dar city, 2014

Factors	HIV status		COR (95% CI)	AOR (95% CI)	P-value
	Positive n (%)	Negative n (%)			
Sex					
Female	6 (3.5%)	165 (96.5%)	1		
Male	8 (3.8%)	203 (96.2%)	0.92 (0.31–2.71)	–	–
Age (years)					
21–30	6 (2.8%)	206 (97.2%)	1	1	
31–40	5 (10.4%)	43 (89.5%)	*3.99* (*1.17–13.68*)	1.78 (0.41–7.67)	0.440
41–50	3 (17.6%)	14 (82.4%)	*7.36* (*1.66–32.58*)	2.55 (0.46–14.1)	0.283
Marital status					
Single	2 (0.7%)	276 (99.3%)	1		
Married	8 (13.3%)	52 (86.7%)	*21.23* (*4.38–102.82*)	*7.92* (*1.32–47.31*)	*0.023*
Divorced/widowed	4 (9.1%)	40 (90.1%)	*13.8* (*2.45–77.8*)	2.35 (0.31–17.65)	0.405
Residence					
Urban	12 (4%)	290 (96%)	1		
Rural	2 (2.5%)	78 (97.5%)	1.61 (0.35–7.36)	–	–
Condom use					
Yes	3 (3%)	98 (97%)	1	1	
No	11 (9.9%)	100 (90.1%)	3.59 (0.97–13.27)	3.24 (0.69–15.28)	0.137
History of blood receiving					
No	13 (3.4%)	365 (96.6%)	1	1	
Yes	1 (25%)	3 (75%)	9.36 (0.91–96.17)	1.24 (0.04–36.7)	0.900
History of tattooing					
No	9 (2.7%)	323 (97.3%)	1		
Yes	5 (10%)	45 (90%)	*3.99* (*1.28–12.43*)	3.02 (0.68–13.5)	0.148
History of hospital admission					
No	11 (3.2%)	337 (96.8)	1		
Yes	3 (8.8%)	31 (91.2%)	2.96 (0.78–11.19)	1.34 (0.24–8.06)	0.707
No of sexual partner					
≤ 1	6 (2.1%)	282 (97.9%)	1	1	
≥ 2	8 (8.5%)	86 (91.5%)	*4.37* (*1.48–12.94*)	1.19 (0.28–5.15)	0.813
Ulcer					
No	13 (3.5%)	361 (96.5%)	1	1	
Yes	1 (12.5%)	7 (87.5%)	3.97 (0.45–34.64)	2.45 (0.19–30.78)	0.489

Significant values are in italics

Age group and marital status were significant associate with HCV infection in bivariate analysis, but only age group the participants were continue significantly associate with HCV infection during multivariate analysis. The age group 41–50 years old (AOR = 65.65; 95% CI 4.57–943, P = 0.002) was found much higher risk for HCV infection than age group of 21–30 years. All HCV antibody positive study subjects had no history of blood transfusion, surgery, sexual transmitted diseases, liver diseases and sharing of sharp materials (Table 3).

**Table 3:** Bivariate and multivariate analysis of risk factors for HCV infection among VCT clients attending Private health facilities in Bahir Dar city, 2014

Factors	HCV status		COR (95% CI)	AOR (95% CI)	P-value
	Positive N (%)	Negative N (%)			
Age (years)					
21–30	3 (1.4%)	209 (98.6%)	1	1	
31–40	1 (2.1%)	47 (97.9%)	1.48 (0.15–14.57)	1.69 (0.14–20.12)	0.680
41–50	4 (23.5%)	13 (76.5%)	*21.44* (*4.33–106*)	*65.65* (*4.57–943*)	*0.002*
Marital status					
Single	4 (1.4%)	274 (98.6%)	1		
Married	1 (1.7%)	59 (98.3%)	1.16 (0.13–10.58)	0.08 (0.00–1.95)	0.122
Divorced/widowed	3 (6.8%)	41 (93.2%)	*5.01* (*1.08–23.21*)	0.5 (0.04–6)	0.580
Education level					
Un able to read and write	3 (7.7%)	36 (92.3%)	1	1	
Grade (1–8th)	2 (1.4%)	136 (98.6%)	0.18 (0.28–1.1)	0.39 (0.04–3.7)	0.409
Grade (9–12th)	1 (0.9%)	114 (99.1%)	0.11 (0.01–1.04)	0.14 (0.01–2)	0.150
Above grade 12th	2 (2.2%)	88 (97.8%)	0.27 (0.27–1.7)	0.4 (0.05–5)	0.538
Tooth extraction					
No	7 (2.0%)	340 (98%)	1		
Yes	1 (2.9%)	34 (97.1%)	1.43 (0.17–11.96)	–	–
No. of sexual partner					
≤ 1	7 (2.4%)	281 (98.7%)	1		
≥ 2	1 (1.1%)	93 (98.9%)	0.43 (0.05–3.56)	–	–
Hospital admission					
No	7 (2.0%)	341 (98%)	1		
Yes	1 (2.9%)	33 (97.1%)	1.48 (0.18–12.38)	–	–

Significant values are in italics

*COR* crude odds ratio, *AOR* adjusted odds ratio, *CI* confidence interval

### Discussion

This study reveals that overall prevalence of HIV infection among VCT clients was 3.7%. The result is consistent with studies conducted in Gondar among blood donors (3.8%) [8], Bahir Dar among couples (3.6%) [9] and Nigeria among pregnant women (3.0%) [10]. On the other hand, this finding is higher compared with EDHS 2011 data (1.5%) in the general population [6] and among blood donors in Jimma (2.1%) [11]. Clients coming to VCT service might have higher risk behavior and it will expose them for HIV infection which can lead to greater prevalence. In contrast, it is lower than studies conducted from other African countries like Nigeria among VCT clients (12%) [12], Cameron in the general population (7.4%) [13] and Gambia in the general population (6.7%) [14]. The discrepancy might be due to variable degree of awareness about HIV infection transmission among the study subjects.

With regard to factors associated with HIV, the current stud results showed that married study participant’s had significantly associated with HIV infection (P value < 0.05). Unlikely study conducted in Bahir Dar showed that single individuals (premarital couples) were more affected with HIV than married individuals. This may be explained by in Ethiopia there is a marriage season and most of people married in this season. Even though, the current data had taken during this season, most of VCT clients who were single (premarital couples) came for further checkup in our study site after they checked their HIV status previously and this might be the reason HIV infection more prevalent among married group than single study participant’s.

The overall prevalence of HCV among VCT clients was 2.1%. This finding is in line with the result from a survey conducted in Ethiopia (2.0%) [7] and Gambia in the general population (2.1%) [14]. Moreover, it is comparable with reports in Ethiopia among healthy volunteer blood donors (1.4%) [15], Deber Markos among VCT clients (1.4%) [16] and Mekelle among HIV negative study subjects (1.6%) [17]. On the contrary, this finding is higher as compared to a survey conducted in Addis Ababa (0.9%) [18], Gondar among blood donors (0.7%) [8] and Gondar among medical west handlers (1.0%) [19]. This disparity might be due to variable degree of exposure to HCV risk factors among the study subjects.

In this study, all HCV positive study participants were found males. Studies conducted in Cameron and Gambia identified that anti-HCV antibody positivity was significantly higher among males [15, 16]. This higher HCV prevalence in males might be due to frequent exposure to higher risk behavior in comparison to females and are therefore more prone to HCV transmission.

The sero-prevalence of HCV has increased as age of participants increased and it was significantly higher in the age group of 41–50 years. This is similarly observed in a survey of HCV prevalence conducted in Ethiopia in 1993 [8]. A study conducted in southern Iran also confirmed that the rate of HCV positivity increased with increasing age and significantly highest HCV positivity was found in the 40–49 age group [20]. It is possible that older age have lived most of their lives exposed to potential risk factors, such as the transfusion of contaminated blood products and medical and therapeutic procedures performed without standard precautionary measures.

### Conclusions

In this study, only married study subjects had significantly associated with HIV infection. The highest proportion of both HIV and HCV infections were found in the age group 41–50 years old. HCV and HIV co-infection was not found among VCT clients in this study. Further large scale research is required to elaborate potential factors associated of HCV and HIV infections.

## Limitations of the study

Numbers of HCV and HIV positive cases are too small to draw significant association with the exposure status of participants to various potential risk factors. In addition HCV viral loads were not performed.

## Data Availability

The finding of this study is generated from the data collected and analyzed based on the stated methods and materials. All data are already found in the manuscript and there is no supplementary file. The original data supporting this finding will be available at any time upon request.

## Abbreviations

EDHS: Ethiopian Demography Health Survey
HCV: Hepatitis C virus
HIV: human immunodeficiency virus
VCT: voluntary counseling and testing
